# Rules Based Data Quality Assessment on Claims Database

**DOI:** 10.3233/SHTI200567

**Published:** 2020-06-26

**Authors:** Mary A. GADDE, Zhan WANG, Meredith ZOZUS, John B. TALBURT, Melody L. GREER

**Affiliations:** a University of Arkansas for Medical Sciences, Littlerock, Arkansas, USA; b University of Texas Health Science Center, San Antonio, TX, USA

**Keywords:** Healthcare Rules, Data quality

## Abstract

Data quality problems in coded clinical and administrative data have persisted ever since diagnoses and procedures were first coded and used for healthcare billing. These data are used in clinical decision-making introducing a route for iatrogenesis. As we share data on regional Health Information Exchanges (HIEs) and include them in electronic health records the potential for harm may be increased. To study this problem we applied rules-based data quality checks that have been previously tested on Electronic Health Records (EHR) data on a limited set of aggregated claims data. Medicaid claims data was used exclusively. CMS has clear guidelines for claims submitted for Medicaid patients and penalties are incurred for erroneous claims, which should ensure a high quality data source, however reports of low and varying sensitivity, specificity, positive and negative predictive value of coded diagnoses are common. To identify data quality defects in claims data in a state All Payer Claims Dataset (APCD) we applied and evaluated a recently developed rules-based data quality assessment and monitoring system for Electronic Health Record (EHR) data to test effectiveness in claims data. These rules, that are feasible for “All Payer Claims data” and Medicaid data are identified, applied and the Data Quality issue results are produced.

## 1. Introduction

APCD data are large State databases that include medical claims, pharmacy claims, dental claims, and eligibility and provider files collected from private and public payers. Claims data provide information on how and where health care dollars are spent; it provides price transparency; health care quality; and payment reform evaluation.[1,2] This information is used to determine the rate of health care inflation, an important outcome of health care reform efforts. [2] Rules-based data quality assessment, though common in clinical research, is not often applied in healthcare facilities.[4] A large system leveraging a knowledge base of over 63,000 rules was developed and tested in healthcare data. In this work, a rules-based approach to data error identification was explored through compilation of over 6,000 data quality rules. The rules were categorized based on topic and logic yielding twenty-two rule templates and associated knowledge tables (hereafter referred to as the Rules) used by the rule templates.[4] Given common use of such rules by payers in the claims process, we posited that claims data would contain few, if any of these discrepancies. However, the limitations and inaccuracy in claims data has been consistently reported in the literature since the initial assessments of claims data. [5] Nine out of twenty-two rules developed and implemented by Wang et. al. (2019) [4] are relevant to data elements in APCD data.

## 2. Methods

Because of voluminous APCD data, only records from 2017 were subjected to the nine rules. Most of the other rules are related to Clinical data and thus not feasible to apply on APCD. The rules are to find data quality problems, for example, incompatibility, incompleteness, and data value out of range. Integrity rules are defined by database constraints and column data types. The two rules for age parameter: (1) Age and Diagnosis (incompatibility) 2. Age and Procedure (incompatibility).

Only APCD Medicaid records are taken. The total number of patients in APCD data is calculated based on the number of encounters, i.e. service start date to service end dates.

**Rule 1, 2** are based on Age and diagnosis/procedure. For Rule_Age_Dx which is Age with Diagnosis incompatibility rule, three categories are defined for this rule. They are (1) Only applicable to maternity patients aged 12 – 55 years inclusive (2) Only applicable to newborns of age 0 years (3) Only applicable to pediatric patients aged 0 – 17 years inclusive. ICD codes for these categories are mapped with APCD Medicaid records and the number of invalid patient counts for APCD Medicaid are given as result set. For **Rule 2,** i.e. Age with procedure Rule_Age_Px, different categories of valid begin and end age are given along with their Current Procedural Terminology (CPT) codes. APCD data is validated based on the valid begin and end age mapping with the date of birth of the patients and the overall invalid patient counts are given in result set. **Rule 3, 4** are based on Gender and diagnosis/procedure. For Rule_Gender_Dx Gender with diagnosis incompatibility rule, gender is verified by mapping ICD codes of the Rule with APCD Medicaid codes The number of Invalid gender counts are collected, the same steps are followed for gender with procedure rule Rule_Gender_Px and the number of patients with invalid counts for APCD is collected **Rule 5** refers to gender and clinical specialty (incompatibility). The clinical specialties are narrowed to Maternal-Fetal Medicine and Obstetrics & Gynecology. The invalid gender for these records is male. The number of patients with invalid gender and clinical specialty is collected for this Rule. In **Rule 6** InPatient Only (IPO) Procedure *Admission date, hour, type* and the *discharge date* fields are taken into consideration to find invalid patient counts. The ICD codes that are required for inpatients are mapped with APCD records and the number of patients with invalid *Inpatient only feature are taken.* The last three rules (**Rule 7** Demographics data elements, **Rule 8** Time Sequence, **Rule 9** Date In Future) are focused primarily on clinical data and few of the categories can be customized for APCD data. Based on this, the validation of records was done by taking *Date of birth* and *Death* dates fields. For sequence of events, *Date of birth*, A*dmit date*, *Date of encounter*, *Claim processed dates*, *Diagnosis* and *procedure dates*, *Discharge dates* fields were considered.

## 3. Results

Total number of patients in APCD data is 52,185. For **Rule1:** Age_Dx - There are 88 patients that have ICD codes matching with ICD codes of Rule1 and these 88 patients have 175 encounters or visits to the medical center, the last column HAC (Adverse Events) of Table 1 reflects the number of patients with Adverse Events-Human Acquired Conditions (HAC) ICD codes, that are similar to ICD codes for Rule1.

## 4. Conclusions

This work demonstrates the value of using data quality rules developed for EHR data on claims data for error identification. Both claims and EHR data are collected for administrative purposes but they differ in their focus and use. While EHR data mirrors the decisions and practice of clinicians, claims data exists to support health plan coverage decisions. This results in two perspectives on the same information. Because the APCD is mainly claims data, nine rules were identified and applied from a set of 22 possible. The remaining rules test clinical values that are not present in claims data. The resulting set of patients with the encounters, instead of an overall count of encounters, is an important data preparation step for data quality evaluation. We have demonstrated that rule based data quality assessment identifies real problems and using these rules to test the merit of claims data appears to be feasible. While there is significant additional work to be done in this area, the exploration of the rule template and associated knowledge base tables for EHR data on claims data shows the approach to be successful, the number of rules likely tractable, and their management scalable.

## Figures and Tables

**Table 1.** Results of Rules applied on APCD Medicaid data

Rules with AR (Medicaid)	Rules on Medicaid data and Claims data for Number of patients	Encounters	APCD	Adverse Events
Rule 1	Age and Diagnosis (incompatibility) - total patients	175	88	0
	Number of patients with Invalid Age and with Diagnosis	5	4	0
Rule 2	Age and Procedure (incompatibility) - total patients	0	0	0
	Number of patients with Invalid Age and with Procedure	0	0	0
Rule 3	Gender and Diagnosis (incompatibility) - total patients	8549	3622	0
	Total number of male and female invalid patients	459	296	0
Rule 4	Gender and Procedure (incompatibility)-Overall patients	0	0	0
Rule 5	Gender and clinical specialty (incompatibility) - Total patients	6	3	3
	Number of invalid records for Gender and clinical specialty	0	0	0
Rule 6	In Patient Only(IPO) Procedure (incompatibility) - Total patients	0	0	0
Rule 7	Demographics data elements (value out of range) - total patients	0	0	0
Rule 8	Time Sequence	0	0	0
Rule 9	Date in the future	0	0	0
